# Growing Old

**Published:** 1887-02-19

**Authors:** 


### GROWING OLD.

BY A COTTAGE HOSPITAL NURSE.

I SIT alone in the twilight,
In the rosy firelight glow,
And my thoughts have wandered far away
To the days of long ago,—
To the days when all the future
Was wrapped in a golden haze,
When the present brought only sunshine,
Those happy summer days;
When my heart was young and eager,
For right, and love, and truth,
And faith in human goodness
The priceless boon of youth.
Why was the golden sunshine
Brighter in days of yore?
Where is the joy in simple things
That comes to us no more?
The sun shines bright as ever,
But my eyes are dim with tears,
And voices from a vanished past
Are sounding in my ears;
Betwixt me and my vanished youth
Long years of shadow lie;
My hand and heart are weary now,
And the end is drawing nigh.
Oh! you, who mourn for vanished youth,
That can never come again;
Oh! you, who have proved this poor world's best
To be fraught with grief and pain,—
Are you not glad at the weary end
To lay your burdens down?
Are you not glad to change the cross
For a bright and glorious crown?
Do you not dream of the perfect life
That holds nor grief nor pain?
Do you not think at the feet of God
You will find your youth again?—
Your youth with a new sweet beauty,
The youth of a fair new life,
Life in its full completeness
After death, and sin, and strife?
And the seed you sowed with weeping
Shall turn to golden grain,
Your buried hopes and love and joys
You shall find them all again.
In the fulness of that perfect life
Nor love nor joys grow cold,
In the heart of God, in the home above,
There is no growing old.

